# Supplementary material for: Intimacy and sexual functioning after cancer: The intersection with psychological flexibility
Source: PLOS Ment Health. 2024 Jun 12;1(1):e0000001. doi: 10.1371/journal.pmen.0000001 (PMC12798418; doi:10.1371/journal.pmen.0000001)
Supplement: S1 File — (DOCX) [file pmen.0000001.s001.docx]

**SUPPLEMENTARY INFORMATION**

**Contents**

[QUALITY OF LIFE IN ADULT CANCER SURVIVORS SCALE 2](#_Toc163476673)

[PERSONAL ASSESSMENT OF INTIMACY IN RELATIONSHIPS (PAIR) 3](#_Toc163476674)

[COMPREHENSIVE ASSESSMENT OF ACCEPTANCE AND COMMITMENT THERAPY (CompACT) 4](#_Toc163476675)

[PATIENT HEALTH QUESTIONNAIRE MOOD SCALE (PHQ-9) 5](#_Toc163476676)

[GENERALIZED ANXIETY DISORDER (GAD-7) 6](#_Toc163476677)

[SATISFACTION WITH LIFE 7](#_Toc163476678)

[CHANGES IN SEXUAL FUNCTIONING QUESTIONNAIRE SHORT-FORM 8](#_Toc163476679)

[INTERPERSONAL EXCHANGE MODEL OF SEXUAL SATISFACTION 9](#_Toc163476680)

**QUALITY OF LIFE IN ADULT CANCER SURVIVORS SCALE**Avis, N. E., Smith, K. W., McGraw, S., Smith, R. G., Petronis, V. M., & Carver, C. S. (2005). Assessing quality of life in adult cancer survivors (QLACS). *Quality of life research*, *14*(4), 1007-1023.

**Instructions:** We’d like to ask you about some things that can affect the quality of people’s lives. Some of these questions may sound similar, but please be sure to answer each one. Below is a scale ranging from never to always.

Please indicate how often each of these statements has been true for you in the past four weeks.

**Items are rated on a 7-point scale ranging from Never (1) to Always (7)**

1. You had the energy to do the things you wanted to do.
2. You had difficulty doing activities that require concentrating.
3. You were bothered by having a short attention span.
4. You had trouble remembering things.
5. You felt fatigued.
6. You felt happy.
7. You felt blue or depressed.
8. You enjoyed life.
9. You worried about little things.
10. You were bothered by being unable to function sexually.
11. You didn’t have energy to do the things you wanted to do.
12. You were dissatisfied with your sex life.
13. You were bothered by pain that kept you from doing the things you wanted to do.
14. You felt tired a lot.
15. You were reluctant to start new relationships.
16. You lacked interest in sex.
17. Your mood was disrupted by pain or its treatment.
18. You avoided social gatherings.
19. You were bothered by mood swings.
20. You avoided your friends.
21. You had aches or pains.
22. You had a positive outlook on life.
23. You were bothered by forgetting what you started to do.
24. You felt anxious.
25. You were reluctant to meet new people.
26. You avoided sexual activity.
27. Pain or its treatment interfered with your social activities.
28. You were content with your life.

## **PERSONAL ASSESSMENT OF INTIMACY IN RELATIONSHIPS (PAIR)**

Schaefer, M. T., & Olson, D. H. (1981). Assessing intimacy: The PAIR inventory. *Journal of marital and family therapy*, *7*(1), 47-60.

**Instructions:** In the first phase please respond to each question as your relationship is now. In the second phase please respond to each question as you would like your relationship to be. Please use the scale below:

**Items are rated on a 6-point scale ranging from Does not describe me/my relationship at all (1) to Describes me/my relationship very well (6)**

1. My partner listens to me when I need someone to talk to.
2. We enjoy spending time with other couples.
3. I am satisfied with our sex life.
4. My partner helps me clarify my thoughts.
5. We enjoy the same recreational activities.
6. My partner has all the qualities I’ve ever wanted in a mate.
7. I can state me feelings without him/her getting defensive.
8. We usually “keep to ourselves.”
9. I feel our sexual activity is just routine.
10. When it comes to having a serious discussion it seems that we have little in common.
11. I share very few of my partners’ interests.
12. There are times when I do not feel a great deal of love and affection for my partner.
13. I often feel distant from my partner.
14. We have very few friends in common.
15. I am able to tell my partner when I want sexual intercourse.
16. I feel “put-down” in a serious conversation with my partner.
17. We like playing together.
18. Every new thing that I have learned about my partner has pleased me.
19. My partner can really understand my hurts and joys.
20. Having time together with friends is an important part of our shared activities.
21. I “hold back” my sexual interest because my partner makes me feel uncomfortable.
22. I feel it is useless to discuss some things with my partner.
23. We enjoy the out-of-doors together.
24. My partner and I understand each other completely.
25. I feel neglected at times by my partner.
26. Many of my partner’s closest friends are also my closest friends.
27. Sexual expression is an essential part of our relationship.
28. My partner frequently tries to change my ideas.
29. We seldom find time to do fun things together.
30. I don’t think anyone could possibly be happier than my partner and I when we are with one another.

## **COMPREHENSIVE ASSESSMENT OF ACCEPTANCE AND COMMITMENT THERAPY (CompACT)**

Francis, A. W., Dawson, D. L., & Golijani-Moghaddam, N. (2016). The development and validation of the Comprehensive assessment of Acceptance and Commitment Therapy processes (CompACT). *Journal of contextual behavioral science*, *5*(3), 134-145.

**Questions are rated on a 7-point scale ranging from strongly disagree (1) to strongly agree(7)**

1. I can identify the things that really matter to me in life and pursue them
2. One of my big goals is to be free from painful emotions
3. I rush through meaningful activities without being really attentive to them
4. I try to stay busy to keep thoughts or feelings from coming
5. I act in ways that are consistent with how I wish to live my life
6. I get so caught up in my thoughts that I am unable to do the things that I most want to do
7. I make choices based on what is important to me, even if it is stressful
8. I tell myself that I shouldn’t have certain thoughts
9. I find it difficult to stay focused on what’s happening in the present
10. I behave in line with my personal values
11. I go out of my way to avoid situations that might bring difficult thoughts, feelings, or sensations
12. Even when doing the things that matter to me, I find myself doing them without paying attention
13. I am willing to fully experience whatever thoughts, feelings and sensations come up for me, without trying to change or defend against them
14. I undertake things that are meaningful to me, even when I find it hard to do so
15. I work hard to keep out upsetting feelings
16. I do jobs or tasks automatically, without being aware of what I'm doing
17. I am able to follow my long terms plans including times when progress is slow
18. Even when something is important to me, I’ll rarely do it if there is a chance it will upset me
19. It seems I am "running on automatic" without much awareness of what I'm doing
20. Thoughts are just thoughts – they don’t control what I do
21. My values are really reflected in my behaviour
22. I can take thoughts and feelings as they come, without attempting to control or avoid them
23. I can keep going with something when it’s important to me

## **PATIENT HEALTH QUESTIONNAIRE MOOD SCALE (PHQ-9)**

Kroenke, K., Spitzer, R. L., & Williams, J. B. (2001). The PHQ‐9: validity of a brief depression severity measure. *Journal of general internal medicine*, *16*(9), 606-613.

**Instructions:** Over the last 2 weeks, how often have you been bothered by any of the following problems?

**Items are rated on a 4-point scale ranging from not at all (0) to over half of the days (3)**

1. Little interest or pleasure in doing things
2. Feeling down, depressed, or hopeless
3. Trouble falling or staying asleep, or sleeping too much
4. Feeling tired or having little energy
5. Poor appetite or overeating
6. Feeling bad about yourself – or that you are a failure or have let yourself or your family down
7. Trouble concentrating on things, such as reading the newspaper or watching television
8. Moving or speaking so slowly that other people could have noticed? Or the opposite – being so fidgety or restless that you have been moving around a lot more than usual
9. Thoughts that you would be better off dead or of hurting yourself in some way

## **GENERALIZED ANXIETY DISORDER (GAD-7)**

Spitzer, R. L., Kroenke, K., Williams, J. B., & Löwe, B. (2006). A brief measure for assessing generalized anxiety disorder: the GAD-7. *Archives of internal medicine*, *166*(10), 1092-1097.

**Instructions:** Over the last 2 weeks, how often have you been bothered by any of the following problems?

**Items are rated on a 4-point scale ranging from not at all (0) to over half of the days (3)**

1. Feeling nervous, anxious or on edge
2. Not being able to stop or control worrying
3. Worrying too much about different things
4. Trouble relaxing
5. Being so restless that it is hard to sit still
6. Becoming easily annoyed or irritable
7. Feeling afraid, as if something awful might happen

## **SATISFACTION WITH LIFE**

Diener, E. D., Emmons, R. A., Larsen, R. J., & Griffin, S. (1985). The satisfaction with life scale. *Journal of personality assessment*, *49*(1), 71-75.

**Instructions:** Below are five statements that you may agree or disagree with.

**Using the 1 (strongly disagree) to 7 (strongly agree) scale below, indicate your agreement with them by selecting the appropriate number**. Please be open and honest in your responding.

1. In most ways my life is close to my ideal.
2. The conditions of my life are excellent.
3. I am satisfied with my life.
4. So far I have gotten the important things I want in life.
5. If I could live forever I would change almost nothing.

## **CHANGES IN SEXUAL FUNCTIONING QUESTIONNAIRE SHORT-FORM**

Keller, A., McGarvey, E. L., & Clayton, A. H. (2006). Reliability and construct validity of the Changes in Sexual Functioning Questionnaire short-form (CSFQ-14). *Journal of Sex & Marital Therapy*, *32*(1), 43-52.

**Instructions:**   Please answer each of the questions, using the rating scale.

**Items are rated on a 5-point scale ranging from never (0) to always (5)**

**FEMALE VERSION**

1. Compared with the most enjoyable it has ever been, how enjoyable or pleasurable is your sex life right now?
2. How frequently do you engage in sexual activity (sexual inter-course, masturbation, etc.) now?
3. How often do you desire to engage in sexual activity?
4. How frequently do you engage in sexual thoughts (thinking about sex, sexual fantasies) now?
5. Do you enjoy books, movies, music or artwork with sexual content?
6. How much pleasure or enjoyment do you get from thinking about and fantasizing about sex?
7. How often do you become sexually aroused?
8. Are you easily aroused?
9. Do you have adequate vaginal lubrication during sexual activity (get wet)?
10. How often do you become aroused and then lose interest?
11. How often do you experience an orgasm?
12. Are you able to have an orgasm when you want to?
13. How much pleasure or enjoyment do you get from your orgasms?
14. How often do you have painful orgasm?

**MALE VERSION**

1. Compared with the most enjoyable it has ever been, how enjoyable or pleasurable is your sex life right now?
2. How frequently do you engage in sexual activity (sexual inter-course, masturbation, etc.) now?
3. How often do you desire to engage in sexual activity?
4. How frequently do you engage in sexual thoughts (thinking about sex, sexual fantasies) now?
5. Do you enjoy books, movies, music or artwork with sexual content?
6. How much pleasure or enjoyment do you get from thinking about and fantasizing about sex?
7. How often do you have an erection related or unrelated to sexual activity?
8. Do you get an erection easily?
9. Are you able to maintain an erection?
10. How often do you experience painful, prolonged erections?
11. How often do you have an ejaculation?
12. Are you able to ejaculate when you want to?
13. How much pleasure or enjoyment do you get from your orgasms?
14. How often do you have painful orgasm?

## **INTERPERSONAL EXCHANGE MODEL OF SEXUAL SATISFACTION**

Byers, E. S. (1999). The interpersonal exchange model of sexual satisfaction: Implications for sex therapy with couples. *Canadian Journal of Counselling and Psychotherapy*, *33*(2), 95-111.

**Global Measure of Relationship Satisfaction**
For the following questions, think about how in general you describe your ***overall*** relationship with your partner.

|  | **Very bad** |  |  |  |  | **Very Good** |
| --- | --- | --- | --- | --- | --- | --- |
|  | 1 | 2 | 3 | 4 | 5 | 6 |
|  | **Very unpleasant** |  |  |  |  | **Very pleasant** |
|  | 1 | 2 | 3 | 4 | 5 | 6 |
|  | **Very negative** |  |  |  |  | **Very positive** |
|  | 1 | 2 | 3 | 4 | 5 | 6 |
|  | **Very Unsatisfying** |  |  |  |  | **Very satisfying** |
|  | 1 | 2 | 3 | 4 | 5 | 6 |
|  | **Worthless** |  |  |  |  | **Very Valuable** |
|  | 1 | 2 | 3 | 4 | 5 | 6 |

**Global Measure of Sexual Satisfaction**

For the following questions, think about how in general you describe your ***sexual*** relationship with your partner.

|  | **Very bad** |  |  |  |  | **Very Good** |
| --- | --- | --- | --- | --- | --- | --- |
|  | 1 | 2 | 3 | 4 | 5 | 6 |
|  | **Very unpleasant** |  |  |  |  | **Very pleasant** |
|  | 1 | 2 | 3 | 4 | 5 | 6 |
|  | **Very negative** |  |  |  |  | **Very positive** |
|  | 1 | 2 | 3 | 4 | 5 | 6 |
|  | **Very Unsatisfying** |  |  |  |  | **Very satisfying** |
|  | 1 | 2 | 3 | 4 | 5 | 6 |
|  | **Worthless** |  |  |  |  | **Very Valuable** |
|  | 1 | 2 | 3 | 4 | 5 | 6 |
