# Supplementary material for: Intimacy and sexual functioning after cancer: The intersection with psychological flexibility
Source: PLOS Ment Health. 2024 Jun 12;1(1):e0000001. doi: 10.1371/journal.pmen.0000001 (PMC12798418; doi:10.1371/journal.pmen.0000001)
Supplement: Attachment — Submitted filename: response to reviewers.docx [file pmen.0000001.s002.docx]

Dear Dr. Mahato and Reviewers,

We would like to thank you and the reviewers for the timely review. Our responses to the comments are in the table below and are tracked in the revised manuscript.

We double checked the references using a retracted article database through CrossRef. We also used the function in Zotoro to ensure that none of the references we included had been retracted. No retractions were indicated by either method. If there are retractions, please let us know and we will modify and replace the retractions.

I used PACE to modify our Figure 1 for publication. The uploaded figure is the one that PACE saved.

| Reviewer Comment | Response to Reviewer |
| --- | --- |
| Page 12 and 13 (8), the section on implication of Acceptance and commitment therapy, gives an impression that the participants underwent some therapeutic program and does not match as per research objectives stated in the abstract and is unwarranted. | The headings and section content were changed to reflect the study design more accurately. |
| Conceptualizing description of the psychological variables (Psychological flexibility) may be expanded. Whether it is same or different with the concept of resilience, and how the concept has been assessed in available literature. | More information was added, and a section was created for psychological flexibility.  See pages 7- 10 |
| In methods section please write full name of the tools used in header (ACT process). | Correction made |
| Exclusion criteria if any in brief need to be mentioned. | “All participants who met this inclusion criteria and completed all the measures were included in the analyses.” |
| Page 15- name of rating scale should be Generalized Anxiety Disorder- 7 item | Correction made. |
| Reference 8. check format | We double checked the reference; it was correct – the issue number as formatted was in the original paper citation |
