# Supplementary material for: Intimacy and sexual functioning after cancer: The intersection with psychological flexibility
Source: PLOS Ment Health. 2024 Jun 12;1(1):e0000001. doi: 10.1371/journal.pmen.0000001 (PMC12798418; doi:10.1371/journal.pmen.0000001)
Supplement: Attachment — Submitted filename: Response to Reviewers.docx [file pmen.0000001.s003.docx]

Dear Dr. Mahato and Reviewers,

We would like to thank you and the reviewers for the timely review of our second revision. Our responses to the comments are in the table below and are tracked in the revised manuscript.

| Editor Comment | Response to Editor |
| --- | --- |
| All PLOS journals now require all data underlying the findings described in their manuscript to be freely available to other researchers | We received approval from our Research Ethics Board to include the anonymized data. The data is posted on Open Science Framework and data availability is noted in our paper. The doi is: DOI 10.17605/OSF.IO/FR5QT |
| Please review your reference list to ensure that it is complete and correct. If you have cited papers that have been retracted, please include the rationale for doing so in the manuscript text, or remove these references and replace them with relevant current references. | We double checked the references using a retracted article database through CrossRef. We also used the function in Zotoro to ensure that none of the references we included had been retracted. No retractions were indicated by either method. If there are retractions, please let us know and we will modify and replace the retractions. |
| Abstract: I think you need to include the purpose/aim of this study in the abstract, just before starting to write on methods. I see that you have in the main text but it is also important to summarize it here. | **We included:** Given the lack of research examining how PF affects relationship and sexual satisfaction, our purpose was to elucidate the relationship between variables associated with partner intimacy, pillars of PF, and variables related to psychological wellness. |
| Introduction: This section is well written but it is fairly long (7 pages) making the readers to lose touch with the key message. I advise that the authors should remove the sub-headings under this section but rather write precise background of 2-3pages that talks about everything relating to the topic. You can present your points in paragraph. | We cut down the information in the Introduction; it is not 5 double-spaced pages. Given that Reviewer 1 requested information on Acceptance and Commitment Therapy to be expanded during our first revision, we did not modify that information. We reduced the number of subheadings but felt we needed to keep two major ones in the introduction. |
| Participants: In this section it would be better to stick to describing the participant but you seem to have combine with background findings. Kindly separate this. Background findings must be taken to result section as the first part of result section. | The section was moved to the start of the Results section. |
| You also reported that all respondents were currently in what they defined as a romantic relationship. What was your operational definition of “Romantic relationship” | We added the operational definition as, “For this study, we asked people to indicate their relationship status. Only individuals who reported being in a romantic relationship (in a relationship, married, in a civil partnership/civil union) were eligible to participate.”  We know that this definition is broad but we wanted to capture survivors who reported that they were in a romantic relationship; we were not interested the effect of being in a specific type of relationship (i.e., married) |
| Operational definitions: I suggest that you select out and insert few operational definitions for this study. | We were unclear where to include this information in the manuscript. Our operational definitions for each variable are included in the materials section; for example, the operational definition for satisfaction with life is the scores on the Satisfaction with Life Questionnaire. We can include more information but felt that that information was already included.  In the discussion, we made sure that each construct was tied to the scale used to measure it. For example, for depression, we said depression (measured by PHQ-9 scores) |
| Data Analysis and Management: You need to have this in your method section. I can see that you have described it in the result section | We moved this information to the Methods section. |
| Abbreviations: You have used this abbreviation “e.g.” too many times. I prefer that you write in full. | We used the APA convention regarding abbreviations. We have replaced e.g. with “for example”. |
| Supplementary Materials: Tools used in this study; such as Patient Health Questionnaire, Generalized Anxiety Disorder 7-item, Changes in Sexual Functioning Questionnaire, Satisfaction with Life Scale. All these tools need to be attached as appendix/supplementary materials to support this study. Kindly attach them. | The supplementary information is now attached. |
| Limitations: Document the limitations of your study | This section was added. |
